# A Comparison of Wood Density between Classical Cremonese and Modern Violins

**DOI:** 10.1371/journal.pone.0002554

**Published:** 2008-07-02

**Authors:** Berend C. Stoel, Terry M. Borman

**Affiliations:** 1 Department of Radiology, Division of Image Processing, Leiden University Medical Center, Leiden, The Netherlands; 2 Borman Violins, Fayetteville, Arkansas, United States of America; Purdue University, United States of America

## Abstract

Classical violins created by Cremonese masters, such as Antonio Stradivari and Giuseppe Guarneri Del Gesu, have become the benchmark to which the sound of all violins are compared in terms of their abilities of expressiveness and projection. By general consensus, no luthier since that time has been able to replicate the sound quality of these classical instruments. The vibration and sound radiation characteristics of a violin are determined by an instrument's geometry and the material properties of the wood. New test methods allow the non-destructive examination of one of the key material properties, the wood density, at the growth ring level of detail. The densities of five classical and eight modern violins were compared, using computed tomography and specially developed image-processing software. No significant differences were found between the median densities of the modern and the antique violins, however the density difference between wood grains of early and late growth was significantly smaller in the classical Cremonese violins compared with modern violins, in both the top (Spruce) and back (Maple) plates (p = 0.028 and 0.008, respectively). The mean density differential (SE) of the top plates of the modern and classical violins was 274 (26.6) and 183 (11.7) gram/liter. For the back plates, the values were 128 (2.6) and 115 (2.0) gram/liter. These differences in density differentials may reflect similar changes in stiffness distributions, which could directly impact vibrational efficacy or indirectly modify sound radiation via altered damping characteristics. Either of these mechanisms may help explain the acoustical differences between the classical and modern violins.

## Introduction

For the past 300 years, the violins of Antonio Stradivari (1634–1737) and Giuseppe Guarneri del Gesu (1698–1744) have excelled in molding a many-nuanced sound that seems to better express the intent of composers and musicians. These classical Cremonese violins have become the benchmark to which all violins are compared. Presently, many believe that violin craftsmanship is at its most advanced point since the days of the Cremonese luthiers, and yet instruments produced today do not match the classical instruments in their abilities of expressiveness and projection. It remains unclear what has kept them, for such a long time and through such changing musical needs, as the most sought after.

Research into the production of high quality sound has focused on a wide range of variables, such as the arching design and contours [1], plate thickness [2], the impact of varnish layers [3], [4], as well as the various elements of set-up, such as the angle of the neck, the impact of the fingerboard and the angle of the strings passing over the bridge. Extensive work has been done searching for the ideal wood properties [5]–[9], although none corresponding exactly to known Cremonese wood properties as most tested samples have been of significantly higher median density than those found to be the case in this study.

Tracheid clusters, produced during annual growth cycles of the tree, create the prominent light/dark grain lines in wood. Early growth wood, created during spring, is primarily responsible for water transport and thus is more porous and less dense than late growth wood, which plays more of a structural support role [10], of much more closely packed tracheids. Wood is an orthotropic material, having differing mechanical properties in three directions: along the grain, across the grain, and slabwise (circumferentially) [11]. The differences in density between early and late growth wood may impact the detailed vibrational behavior, either directly or through altered stiffness or damping characteristics due to these variations. The complex three-dimensional shape of the violin body means that vibration within the audio range involves extensional, bending and shear deformations of the wooden plates involving all three directions. Researchers have commented on wood selection preferences based on these differentials [9], although detailed data are lacking on fine instruments. Wood density is difficult and invasive to measure directly, as an isolated part of the instrument, wrapped in a waterproof container, must be immersed in water to estimate its volume, and the density is calculated by dividing its weight by this volume [12]. Furthermore, this technique does not provide data on density differentials. Computed Tomography (CT) has been used by other researchers [13]–[15] primarily for visual analysis, without fully employing its ability to quantify density or density differentials.

Here we examine the wood density of five classical Cremonese violins; three by Giuseppe Guarneri del Gesu and two by Antonio Stradivari, using quantitative CT densitometry, a rapid and non-invasive technique usually applied in a medical setting [16]. The results from these classical violins were compared to those of eight contemporary violins, made by T. Borman, A.T. King and G. Rabut (Table 1), in order to determine whether objective measurements of material properties can explain the historical consensus on the differences in quality of sound between classical Cremonese and modern violins. At the end of this article we will outline in detail our methodology.

**Table 1:** Table of instruments studied.

Classical violins				
*Maker*	*Date*	*Location*	*Instrument name*	*Luminal volume (Liter)*
Giuseppe Guarneri del Gesu	1734	Cremona, Italy	“ex Rode”	1.87
Giuseppe Guarneri del Gesu	1735	Cremona, Italy	“ex Kubelik”	1.78
Giuseppe Guarneri del Gesu	1735	Cremona, Italy	“ex Plowden”	1.83
Antonio Stradivari	1715	Cremona, Italy	“ex Titian”	1.87
Antonio Stradivari	1734	Cremona, Italy	“ex Wilmotte”	2.04
**Modern instruments**				
Terry M. Borman	1995	Salt Lake City, UT, USA	Viola	2.91
Terry M. Borman	2005	Salt Lake City, UT, USA	Violin	1.93
Terry M. Borman	2005	Salt Lake City, UT, USA	Violin	1.92
Terry M. Borman	2006	Fayetteville, AR, USA	Violin	1.81
A. Thomas King	1995	Potomac, MD, USA	Violin	2.04
A. Thomas King	2006	Potomac, MD, USA	Violin	2.03
Guy Rabut	2003	New York, NY, USA	Violin	1.96
Guy Rabut	2003	New York, NY, USA	Violin	1.98

## Results and Discussion

The violins were scanned at Mount Sinai Hospital in New York City, USA, using a multi-detector row CT scanner (Sensation Cardiac 64, Siemens, Germany). These scans produced 3-dimensional data sets of approximately 1200×512×512 voxels for each violin.

A dedicated computer program was developed to automatically detect the superior and inferior surface of the top and back plates. From these surfaces, the local plate thickness, median wood density and density differential were calculated, as discussed below. Additionally, the volume of the sound box (luminal volume) was calculated (Table 1).

### Plate thickness

**Figure 1:**
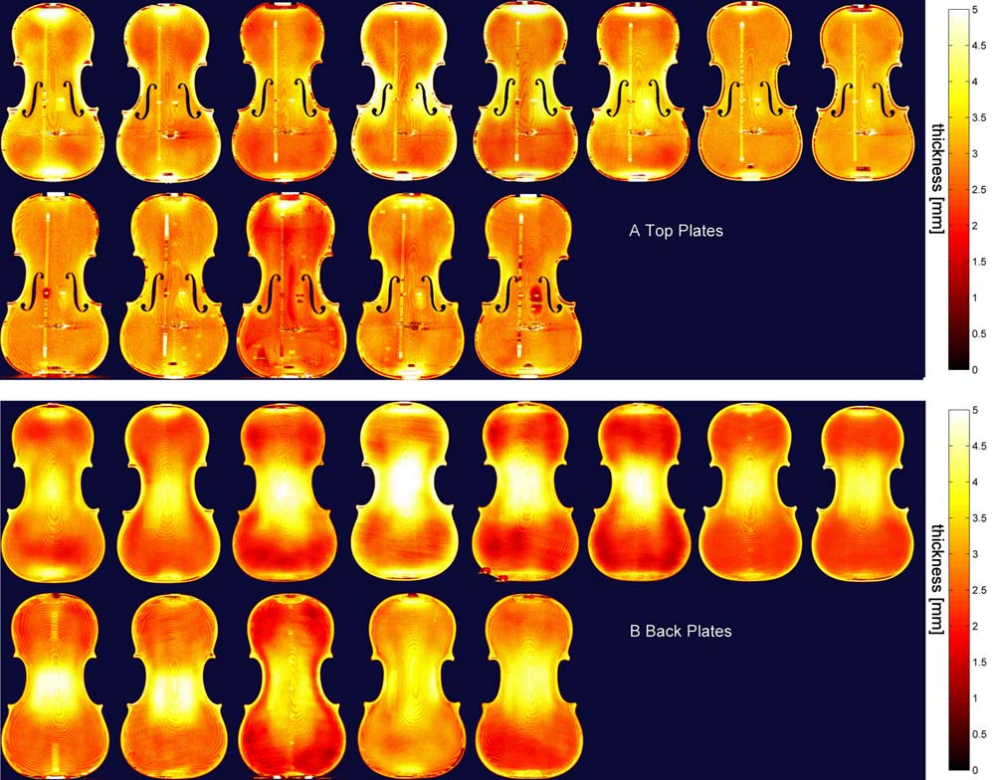
Thickness maps of the top (A) and back plates (B). The contemporary violins are presented on the top row, and the antique on the bottom row. The violins have been anonymised. Scales are given in mm. The fourth instrument on the upper row is a viola, which typically is thicker than a violin (image size has been reduced to match that of the violins).

From the vertical distance between the superior and inferior surface, a thickness map (0–5 mm) was constructed, which represents the plate thickness at each location. Figures 1A and 1B show the thickness maps of the top and back plates, respectively, with the classical violins displayed on the bottom and the modern violins on the top row of the figures. We have adopted the medical model of anonymity. These thickness maps clearly show differences between the violins as well as various repairs. The bass bar could be discerned as a slight thickening in the top plate, since the computer program could not perfectly separate the two wood pieces. The antique plates, with the exception of #3, had very little repair, while resolution was such that even the paper labels with the makers' name could be discriminated (see the rectangular thickening in the back plates, near the left c-bout in Figure 1B). Note that the high X-ray absorption by the metal in the fine tuner on the e-string causes image reconstruction artifacts. The Moiré-like pattern is caused by the somewhat limited resolution of the scanner. Loen [17] has done extensive thickness mapping of violins although a comparative analysis of findings is beyond the purview of this article and our maps are included solely on the basis of the intrinsic link between density and thickness.

### Median density

**Figure 2:**
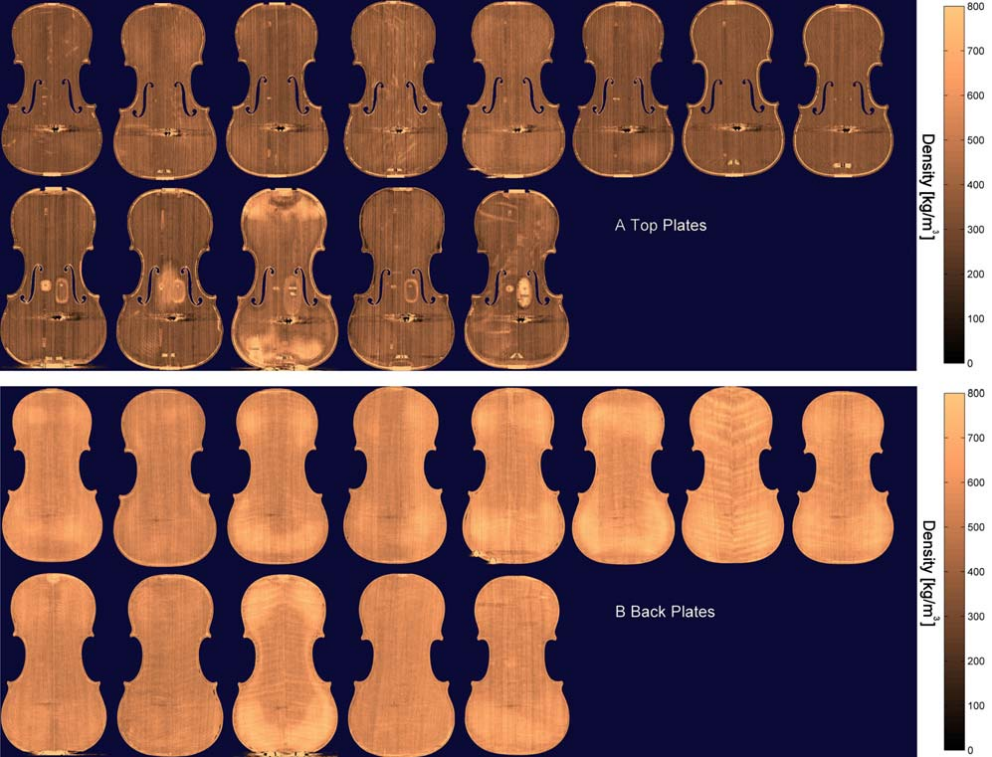
Density maps of the top (A) and back plates (B). The contemporary violins are presented on the top row, and the classical Cremonese on the bottom row. The violins have been anonymised. Scales are given in kg/m3. The central violin in the lower row has had more repair work than the other antique violins as evinced by reduced thickness (Figure 1.) and increased densities. The dark areas at the centre of the lower third of all violin tops are metal artifacts from the string ends. The dependency of the measured density on plate thickness was eliminated in the quantitative analysis.

**Figure 3:**
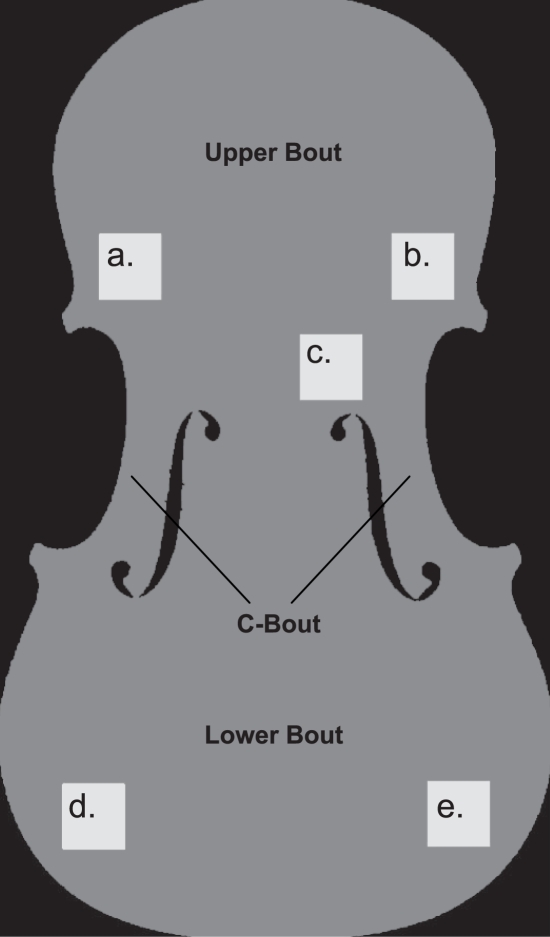
Regions of interest (ROI's) on violin plates. Five different ROI's of 100×100 pixels were defined, carefully avoiding repair work. The same areas were taken from the top and back plates.

The computer program defined an intermediate layer of the violin plates, which was centered exactly between the superior and inferior surfaces. From this intermediate layer, a density map was created, in which the physical density was calculated at each location within the plates. Figures 2A and 2B show the detailed density maps of the top and back plates, respectively. The top and back plates differ in density, as top plates are made from spruce (*Picea abies*) and the rest of the instrument, including the back plate, is made from maple (*Acer Platanoides*). Repair work was clearly visible in the top plates, as indicated by the regions of increased density. Hide glue, used exclusively for violin repair, has a higher density than wood and saturates into the adjacent, undamaged material, thus increasing localized density readings. From this density map, the median density was calculated at five standardized regions of interest (ROI); on the left and right side of the upper and lower bout, and one at the centre (see Figure 3); care was taken to avoid regions of repair work. No significant differences were found between the median densities of the modern and the antique violins (two-tailed Mann-Whitney U test: p = 0.884 and 0.143, for the top and back plate, respectively).

Apart from genetic factors, the overall density of wood is influenced most significantly by the microclimate at the tree's location. A tree growing in a cool area with limited direct solar exposure and little access to water supplies or quality soil will grow slowly and have relatively high overall densities. On the other hand, a tree of the same genetic makeup would grow faster with lower overall densities, if it were located in a more hospitable microclimate, i.e. with adequate solar access, a nutrient laden soil, sufficient quantities of water, a relatively flat local, and without traumatic events causing formation of very dense wood. The former conditions have historically been thought to create high quality tone wood although our findings indicate that the latter conditions will more closely mimic the densities found in this study. As we did not find significant differences in median density between these particular classical and modern violins, these large-scale factors would not be relevant to the sound quality difference between the classical Cremonese and the modern violins.

A violin produces sound by transforming the energy provided by the musician into perturbations of the air. At lower frequencies, below ∼800 Hz, the majority of these waves are produced by the violin acting as a whole. Above this frequency range, specific areas of the instrument vibrate to produce sound. At the current state of understanding, most of these areas are located on the top plate. For this reason, our discussion is primarily focused on spruce wood.

Even after a violin is built, its wood density could vary, since wood is a hygroscopic material and changing relative humidity (due to temperature as well as water vapor levels) would change the measured density. In this context, however, this is not germane, since the studied violins are never exposed to extreme humidity variations due to the conditioned air environments of modern musical settings.

As there was little to no difference in the median wood densities between the modern and the classical Cremonese violins, it may be assumed that modern wood selection practices are similar to those employed in the 1700s.

### Density differentials

In order to determine the amount of late and early growth grains in the wood of each violin plate, we calculated the histogram of densities from each ROI (Figure 3). Wood density may vary each 0.1 mm, which is beyond the resolution of CT. Therefore, a density value of the early and late growth grains could not be determined definitively. A surrogate grain density measure was defined instead by the spread of the bimodal density distribution. The 90^th^ and the 10^th^ percentile points were considered representative of the density of the early and late growth grains, respectively, and the difference between these percentile points was denoted as the ‘density differential’.

**Figure 4:**
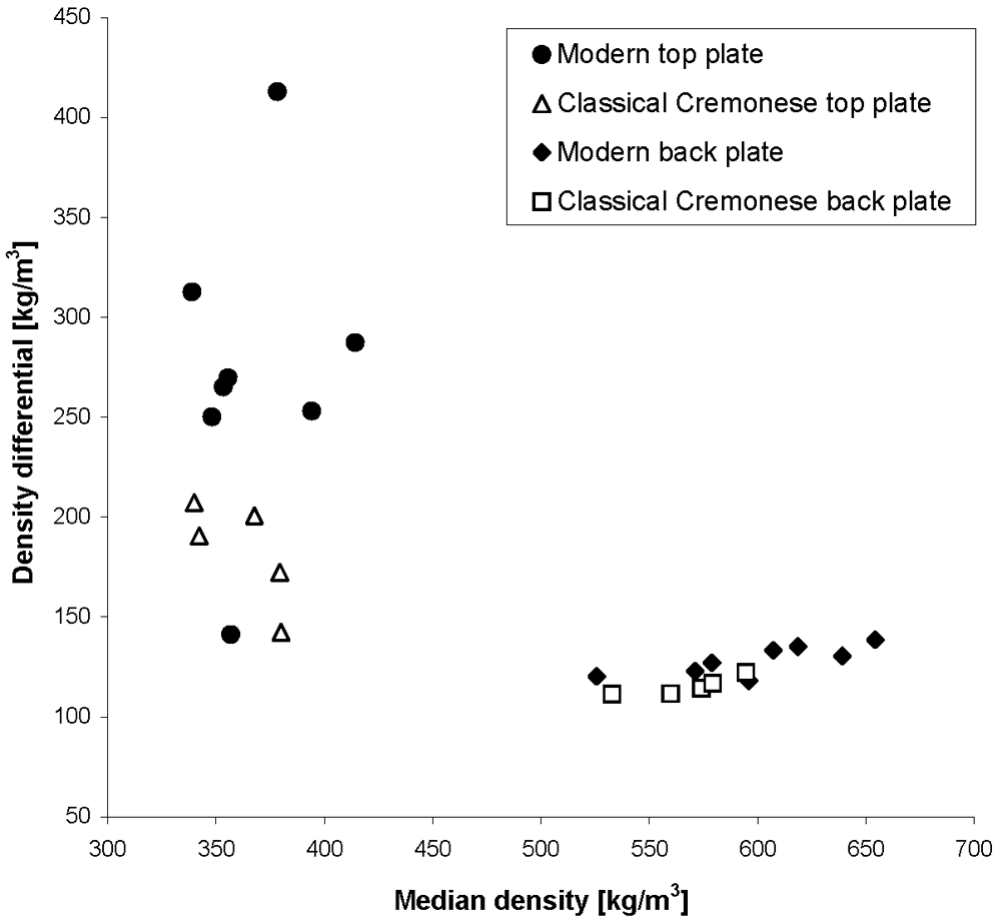
Density differential versus median density of all top and back plates.

In Figure 4, the density differential is plotted against the median density, averaged over all ROIs, which were compared using the two-tailed Mann-Whitney U test. The density differential was significantly lower in the classical Cremonese violins as compared to the modern violins both in the top and back plate (p = 0.028 and 0.008, respectively), meaning that the densities of early and late growth wood were closer together, in the classical violins. The mean density differential (SE) of the top plates of the modern and classical violins were 274 (26.6) and 183 (11.7) gram/liter, respectively. For the back plates, the values were 128 (2.6) and 115 (2.0) gram/liter, respectively. Figure 4 shows four clear “clusters” whereby the wood of the instruments is delineated into two groups: the old and new top plates and the old and new back plates.

Due to the increased repair work on one of the classical instruments, it was necessary to choose the ROI's carefully so as to reflect the true wood density, not that of the repair. In order to realistically compare wood densities, the inclusion criteria for a modern instrument was that the woods were of known European provenance and that they were in a “natural state”, i.e. not treated in any way to alter its material properties. When we noticed the one modern top and back plate of extremely low differential, we contacted the maker who reviewed his records and found that he had acquired these pieces of wood from a supplier who occasionally treated his wood prior to sale. When questioned, the supplier could not be certain if these particular pieces were treated or not. If these plates of unknown origin were removed from the analysis, the differences of the density differential of the top plates between the old and new would be even more striking. In our test pool of spruce tone wood samples we found a similar pattern i.e. new wood having median densities in the same general range and density differentials much higher than that of the Cremonese violins tested.

Spruce density may vary within a tree by as much at 5–8% due to its vertical location within the trunk. Within same tree specimens density is typically lowest between 3 and 6 meters of height. Below 3 meters to ground level there is a slight increase and above 6 meters of tree height density increases in a fairly linear continuum to the apical bud [18]. Since the classical median densities are at the very low end of those found in spruce, this region would provide the closest approximation within individual samples. Additionally, the distance from the pith (centre of the tree) to the perimeter is a well-identified source of density variations within the same tree specimens and in most species, including *Picea abies*, density typically decreases with distance outwards from the pith. This decrease in density has been found to be due to a reduction in early wood density as well as a reduction in late wood proportion and may amount to 15–20% density variations from pith to perimeter [19]. Taken together the north/south (sample height) and east/west (pith to perimeter) localized impacts can amount to an almost 25% density variation within the same tree.

Widths of the individual growth rings are yet another factor influencing wood density that has been well documented to date, although disagreement exists on the quality of this relationship. Growth Ring Width (GRW) in Norway spruce has been shown to have a negative correlation with average density [20] and therefore a non-linear relationship with greater reductions in basic density when the ring widths decrease to 2–3 mm and lesser overall reductions with increasingly wider ring widths. Giordano [21] on the other hand, found a relatively linear relationship for these same parameters. Another study, specifically targeted at violin tone wood [22], did not find a linear relationship and their experimental data pool of 300 samples showed no apparent pattern in density distributions vs. GRW. Their sample ring spacing was however relatively limited, varying only from 0.5 mm to 2 mm, whereas Giordano extended this range to 4 mm (the maximum ring spacing usually found in violins is 2.5 mm to 3 mm; in violas 3 mm to possibly 4 mm and in cellos this can reach 5 mm). Saranpää and Giordano concur that GRW can account for min/max density variability of ∼40%, although arriving at their respective results in different manners [18], [21]. The current state of wood biology delves very little into density differential with the exception of Koubaa [19] using x-ray densitometry to redefine Mork's index (the transition from early wood to late wood).

### Conclusions

The density differentials found in this study may contribute to the generally recognized superior sound production of classical Cremonese violins. Within the violin making tradition there have been many reported ‘secrets’ of the Cremonese makers although usually with little or no supporting documentation. Sporadically, reference is made to the wood treatment referred to as ‘ponding’, whereby wood submerged in stream water (to facilitate transportation or to alter the properties of the wood intentionally) is responsible for the classical Cremonese sound. It has been documented [23] that ponding does alter wood properties significantly, by causing decomposition of various wood elements depending on the particular bacteria or fungus introduced into the wood. Although data on density alteration are not currently available, it is reasonable to assume that this degradation would result in lowered densities; how this impacts density differential would be dependant on the specific treatment. It has been shown that the wood of the classical Cremonese instruments was likely not ponded [24]. However, this does not rule out bacterial or fungal attack as a means of altering new wood to more closely match the material properties of the Cremonese wood. As mentioned earlier, one back and one top plate of the new instruments may have been treated and if this were indeed the case, the treatment used by the supplier would have been ponding. Another technique, referred to as “stewing” wood has been mentioned whereby wood is boiled in different solutions to achieve alterations of density although there is no published data on what this process is actually doing to the wood. Bucur has shown that time plays a role in altering wood properties by decomposition and loss of hemicellulose, thereby resulting in lower density [9] and a priori an alteration of differential, which may also explain our results. Fuming with nitric acid or ammonia are treatments that have been used throughout the years by instrument makers and it is a reasonable assumption that the destructive properties of these agents would lower the density and change the differential depending on which grains, early or late, are most affected. Many other possibilities have been proposed over time, but these are the only ones directly related to density that we are aware of.

In summary, our results clearly document basic material property differences between the woods used by the classical Cremonese and contemporary makers. Although at this point we can do no more than speculate as to the cause, these findings may facilitate replicating the tonal qualities of these ancient instruments.

## Materials and Methods

As CT densitometry depends on a wide range of variables, settings were optimized for the highest sensitivity in distinguishing different wood densities. We analyzed the histograms from four test plates (two top plates and two back plates) and selected the settings, which produced bimodal histograms with the highest separation. The final image acquisition protocol was defined for a multi-detector row CT scanner: 80 kVp, effective mAs of 53, collimation 32×0.6 mm, 1 sec. rotation time, 512×512 matrix, 0.6 mm slice thickness, 0.3 mm increment with a reconstruction filter B50s.

Volumetric analysis was performed with PulmoCMS (Medis Specials BV, Leiden, the Netherlands) and a separate computer program was developed for wood densitometry on a Matlab platform (Matlab, version R2007a, The Mathworks, USA), with its image processing toolbox. The superior and inferior contours were detected in each axial slice by a minimal costs algorithm, using a Sobel edge detector. By stacking all contours, a curved multi-planar reformatted (MPR) image was constructed. No user interaction was needed in the analyses of the violins.

### Validation

Constancy of the CT scanner was monitored using nine test pieces of maple and spruce. The standard deviation of the differences was 7.5 kg/m^3^ (1.8%) and 10.9 kg/m^3^ (4.8%) for the median density and density differential, respectively.

**Figure 5:**
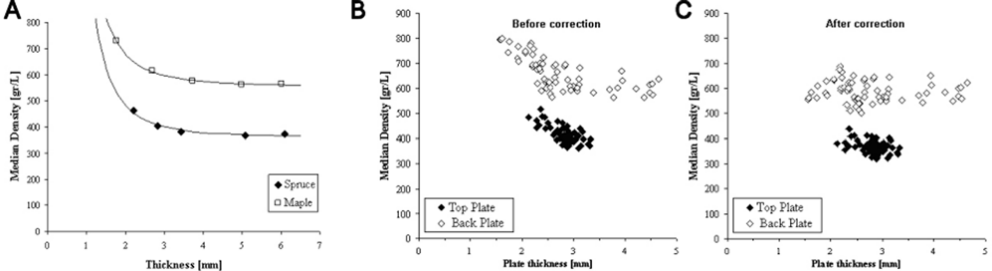
Relation between thickness of the plate and median density. (A) The relation was obtained from the central layer within five spruce and five maple test plates. The curved lines show the mathematical models fitted to this data. (B) The thickness-density relation from the individual ROIs in the violins. (C) The thickness-density relation after correction.

Due to edge enhancement during CT image reconstruction, density values were found to be dependent on plate thickness (as illustrated by comparing Figure 1 and 2 in the main text). Therefore, the presented density values were corrected for thickness, based on measurements from a different sample set of 10 wood samples with thicknesses, ranging from 2 to 6 mm. The measurements were corrected based on a mathematical model, in which the dependency of the median density on plate thickness was estimated (see Figure 5A). The correction was effective, since subsequently no correlation was found between the final density values and the thickness of the plates from all regions of interest (Figure 5B and 5C). As there was no significant difference in plate thickness between the classical and modern violins (Mann-Whitney U test: p = 0.770 an 0.188, for the top and back plate, respectively), plate thickness was not a confounding factor in studying the differences in wood density.

To test the accuracy of the thickness measurements of the plates, the same wood samples were used as in the correction for the thickness dependency. The measured values from CT were compared to the actual thickness measurements using a micrometer on the actual pieces. A small systematic difference was observed of 0.1 mm, which is a fraction of the dimension of one pixel (0.4×0.6×0.6 mm), meaning that plate thicknesses were slightly over-estimated with a constant magnitude, independent of plate thickness.
